# Modeling metabolic homeostasis and nutrient sensing in *Drosophila*: implications for aging and metabolic diseases

**DOI:** 10.1242/dmm.012989

**Published:** 2014-03

**Authors:** Edward Owusu-Ansah, Norbert Perrimon

**Affiliations:** 1 Department of Genetics, Harvard Medical School, 77 Avenue Louis Pasteur, Boston, MA 02115, USA.; 2 Howard Hughes Medical Institute, 77 Avenue Louis Pasteur, Boston, MA 02115, USA.

**Keywords:** Metabolic homeostasis, Nutrient sensing, *Drosophila*

## Abstract

Over the past decade, numerous reports have underscored the similarities between the metabolism of *Drosophila* and vertebrates, with the identification of evolutionarily conserved enzymes and analogous organs that regulate carbohydrate and lipid metabolism. It is now well established that the major metabolic, energy-sensing and endocrine signaling networks of vertebrate systems are also conserved in flies. Accordingly, studies in *Drosophila* are beginning to unravel how perturbed energy balance impinges on lifespan and on the ensuing diseases when energy homeostasis goes awry. Here, we highlight several emerging concepts that are at the nexus between obesity, nutrient sensing, metabolic homeostasis and aging. Specifically, we summarize the endocrine mechanisms that regulate carbohydrate and lipid metabolism, and provide an overview of the neuropeptides that regulate feeding behavior. We further describe the various efforts at modeling the effects of high-fat or -sugar diets in *Drosophila* and the signaling mechanisms involved in integrating organ function. Finally, we draw attention to some of the cardinal discoveries made with these disease models and how these could spur new research questions in vertebrate systems.

## Introduction

Metabolic syndrome – often considered a harbinger of cardiovascular disease – is a complex clinical disorder characterized primarily by abnormal blood lipid levels (dyslipidemia), central obesity, high blood pressure and elevated fasting glucose levels. Although previously considered a debilitating condition restricted to affluent societies, it has now emerged as an issue of major public health significance worldwide. Attempts to uncover therapeutic strategies for alleviating this global phenomenon have focused largely on vertebrate model systems. However, recent observations in *Drosophila* have given credence to the hypothesis that this simple model organism can provide useful information for elucidating the complexities of mammalian metabolism.

*Drosophila* have organ systems that perform essentially the same metabolic functions as their vertebrate counterparts (Leopold and Perrimon, 2007). For instance, there are both oxidative and glycolytic muscles (such as flight and leg muscles, respectively) that consume energy during flight or other forms of locomotion. In addition, the fat body, which stores excess fat as triglycerides (which can be mobilized during times of need using lipases that are orthologous to those found in mammals), functions as the liver and white adipose tissue (Baker and Thummel, 2007; Leopold and Perrimon, 2007). A group of specialized cells, referred to as oenocytes, can function as hepatocytes by mobilizing stored lipids in the fat body during periods of food deprivation (Gutierrez et al., 2007). Moreover, the sophisticated genetic tools available for studies in this organism (del Valle Rodríguez et al., 2011), coupled with its relatively short lifespan, have facilitated the discovery of novel molecules and modes of regulation of multiple aspects of metabolism and aging (Leopold and Perrimon, 2007; Karpac and Jasper, 2009; Alic and Partridge, 2011; Biteau et al., 2011). Here, we highlight recent advances in modeling aspects of metabolic homeostasis in *Drosophila*, especially as it relates to diabetes, obesity and the overall aging process. We begin by highlighting the pathways that regulate normal metabolic homeostasis in *Drosophila*.

## Regulating metabolic homeostasis through the *Drosophila* orthologs of glucagon and insulin

Homeostatic regulation of circulating sugar levels is essential for the health of organisms. For example, impaired fasting glucose (elevated blood sugar) is an important risk factor associated with the development of cardiovascular disease in humans (Kannel et al., 1990). In addition, one of the severe metabolic complications of diabetes is ketoacidosis, which can result from exceptionally high circulating glucose levels (Forbes and Cooper, 2013). In mammals, glucagon and insulin are synthesized in pancreatic α- and β-cells, respectively, with the former largely responsible for breaking down glycogen into sugar, whereas insulin regulates the converse process. Similarly, *Drosophila* produce a glucagon-like peptide, referred to as the adipokinetic hormone (AKH), in a group of neurosecretory cells in the ring gland known as the corpora cardiaca (Kim and Rulifson, 2004; Lee and Park, 2004). Forced AKH expression from the fat body increases trehalose levels (trehalose is the major circulating sugar in *Drosophila*); in contrast, flies devoid of the AKH-producing neurons display a precipitous drop in trehalose levels (Lee and Park, 2004). Nevertheless, the AKH signaling cascade in *Drosophila* is poorly characterized. For instance, other than the ligand (AKH) and receptor (AKH receptor), very little is known about the downstream intracellular kinases and phosphatases, and it is unclear whether there are other ligands and receptors for the pathway. Significantly, because no overt developmental effects are associated with aberrant AKH signaling under non-stressed conditions, it is particularly amenable to genetic screens because data interpretation is unencumbered by alterations in rates of development.

In contrast to AKH signaling, the insulin–insulin-growth-factor signaling (IIS) pathway in *Drosophila* has been more thoroughly characterized. Like many aspects of *Drosophila* metabolism, there are remarkable differences between the effects of insulin signaling during the larval and adult phases. For instance, insulin regulates growth of essentially all tissues during the larval stage but its effect in adults is largely restricted to metabolic homeostasis, resistance to stress, fecundity and lifespan (Broughton et al., 2005; Grönke et al., 2010). There are eight *Drosophila* insulin-like peptides (DILPs) (Table 1), some of which have unique properties and varying tissue and temporal expression patterns (Brogiolo et al., 2001; Ikeya et al., 2002; Rulifson et al., 2002; Broughton et al., 2005; Broughton et al., 2008; Veenstra et al., 2008; Yang et al., 2008; Hsu and Drummond-Barbosa, 2009; Okamoto et al., 2009; Slaidina et al., 2009; Chell and Brand, 2010; O’Brien et al., 2011; Sousa-Nunes et al., 2011; Bai et al., 2012; Colombani et al., 2012; Garelli et al., 2012). Genetic ablation of the insulin-producing cells (IPCs) during early larval stages delays development and results in elevated sugar levels in the larval hemolymph (Rulifson et al., 2002); however, ablation during the adult stage results in reduced fecundity, increased storage of triglycerides and sugars, heightened resistance to starvation and oxidative stress, and prolonged lifespan (Broughton et al., 2005). Nevertheless, because IPCs secrete other peptides as well, it is unclear whether the phenotypes associated with IPC ablation are due solely to the secreted ILPs or other peptides as well.

**Table 1.**
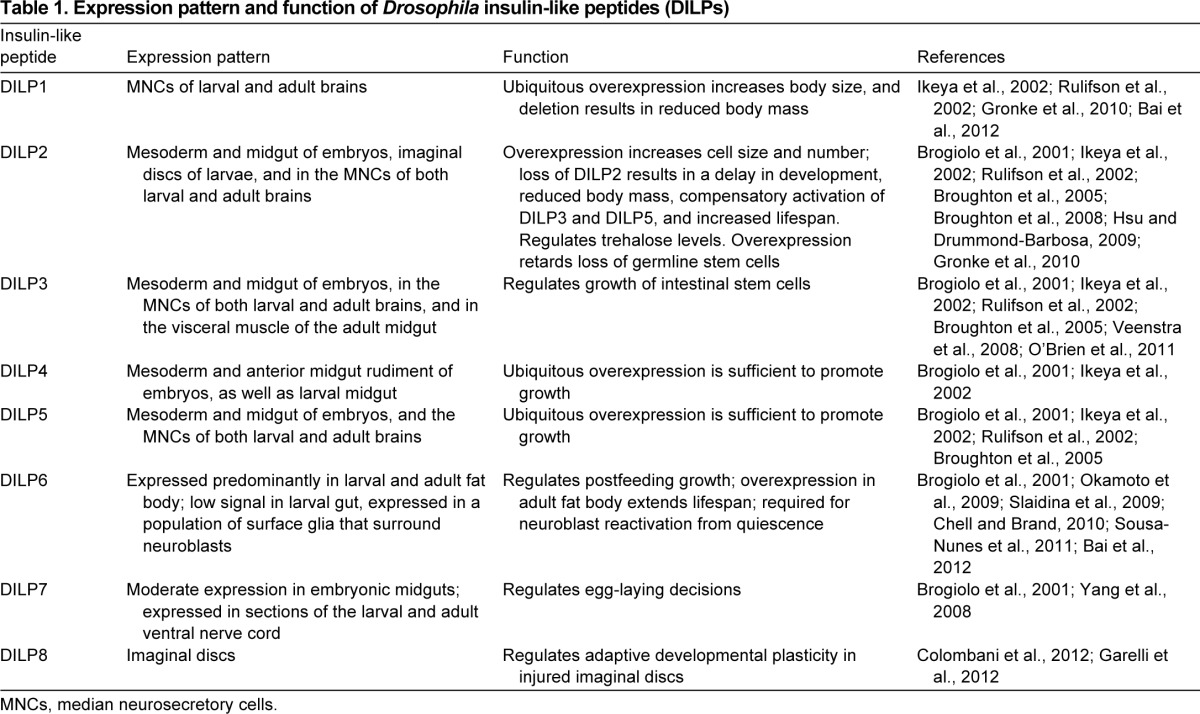
Expression pattern and function of *Drosophila* insulin-like peptides (DILPs)

Although the core intracellular IIS cascade consists of single genes encoding the insulin-like receptor (InR), Akt (protein kinase B), phosphoinositide 3-kinase (PI3K) and forkhead transcription factor FOXO, regulation of insulin signaling in *Drosophila* is proving to be remarkably complex. In addition to the numerous DILPs that regulate insulin signaling (Table 1), there is a growing list of insulin-antagonizing peptides that have notable impacts on insulin signaling. In mammalian systems, insulin signaling can be modulated by a group of secreted insulin growth factor (IGF)-binding proteins (IGFBPs) and IGF-binding related proteins (IGFBP-rPs). Notably, binding of these secretory proteins to IGF can either enhance or suppress insulin signaling by altering the bioavailability of insulin, the rate of degradation of insulin or the ability of insulin to bind to its cognate receptor (Hwa et al., 1999). The importance of IGFBPs is underscored by the fact that IGFBP7 can act as a tumor suppressor (Ruan et al., 2007; Chen et al., 2013). The imaginal morphogenesis protein-Late 2 (Imp-L2), which is a neural/ectodermal development factor in *Drosophila*, has been shown to bind to DILP2 and DILP5 to impair insulin signaling (Alic et al., 2011); however, it promotes insulin signaling in a subset of neurons in the larval brain (Bader et al., 2013). Forced expression of Imp-L2 results in induction of 4E-BP (a marker of insulin repression), causes non-autonomous growth inhibition and triggers many of the phenotypes associated with impaired insulin signaling, such as reduced fecundity, increased triglycerides and extended lifespan (Honegger et al., 2008; Alic et al., 2011). Another insulin-binding peptide is acid labile subunit (ALS), which functions in a trimeric complex with Imp-L2 (Arquier et al., 2008). In addition, secreted decoy of insulin receptor (SDR), which is structurally similar to the extracellular domain of the insulin receptor, has been shown to bind to several DILPs *in vitro* (Okamoto et al., 2013) and, when overexpressed, it upregulates several markers of insulin repression. In addition, overexpression of SDR in larvae produces adult flies that are smaller than controls. SDR and Imp-L2 appear to be mutually exclusive with respect to their ability to bind DILPs. It remains to be tested whether SDR overexpression is sufficient to extend lifespan.

Finally, metabolic homeostasis also requires that organs coordinate their activities by means of the secretion of humoral factors or the propagation of nerve impulses between the organs. In mammalian systems, this is a fairly well-established phenomenon, because the brain is known to process signals relating to the nutritional status of an organism and then elicit appropriate systemic responses. There is mounting evidence that *Drosophila* organs can also ‘communicate’ with each other, and recent studies in flies have unraveled several inter-organ signaling modules, some of which are regulated by DILPs. As a case in point, overexpression of FOXO in flight muscles reduces both feeding behavior and insulin secretion from the IPCs. This in turn delays the accumulation of misfolded protein aggregates not only in muscles, but also non-autonomously in non-muscle tissue (Demontis and Perrimon, 2010).

The fat body has also been shown to remotely control the secretion of DILPs from the IPCs through a mechanism dependent on the ‘target of rapamycin’ (TOR) (Colombani et al., 2003; Géminard et al., 2009). By analyzing a series of elegant *ex vivo* co-cultures of larval brains and fat body tissue, the existence of a humoral signal (or signals) released from the fat body that can impede the secretion of DILPs from the IPCs was proposed (Géminard et al., 2009). Subsequently, unpaired 2 (Upd2), a specific ligand of the *Drosophila* JAK-STAT pathway, was found to be induced in the fat body during the fed state (Rajan and Perrimon, 2012). Interestingly, Upd2 induction in the fat body is also associated with the release of DILPs from the IPCs in the brain (Rajan and Perrimon, 2012). Others have shown that DILP6 was upregulated in the fat body in response to fasting or FOXO overexpression and could also repress secretion of DILP2 from IPCs in the brain (Bai et al., 2012). Importantly, overexpression of DILP6 in head or abdominal fat body caused many of the classic traits associated with downregulation of insulin signaling, such as the presence of increased whole-body triglycerides, stress resistance and lifespan of female flies (Bai et al., 2012). It will be interesting to investigate whether Upd2 and DILP6 act in concert or in parallel to regulate DILP secretion from IPCs.

## Regulation of feeding behavior through peptidergic signaling

A major aspect of metabolic homeostasis is the regulation of feeding behavior. Studies in mammalian systems have shown that the presence of food in the gut stimulates a number of endocrine and neuronal signals that act through complex feedback loops to regulate feeding behavior. A growing list of peptides such as ghrelin, cholecystokinin, glucagon-like peptide-1 (GLP-1) and neuropeptide Y are known to regulate food intake and satiety (Steinert et al., 2013). Interestingly, the overwhelming success of Roux-en-Y gastric bypass surgery, in which the stomach is subdivided and reconnected to the small intestine, in causing significant weight loss in morbidly obese individuals has been linked to alterations in gut peptides that regulate feeding and satiety (Kellum et al., 1990; le Roux et al., 2006; Morínigo et al., 2006). Not surprisingly, there is mounting research to explore the possibility of interfering with the action of these factors as a means to control obesity. For instance, GLP-1, produced primarily in the distal intestine, is rapidly released after a meal and suppresses food intake in several organisms (Turton et al., 1996; Donahey et al., 1998; Chelikani et al., 2005). Accordingly, administration of GLP-1 to subjects with type 2 diabetes for 6 weeks resulted in reduced appetite, significant weight loss and a decrease in plasma glucose levels (Zander et al., 2002). In clinical trials, Exenatide (an agonist of the GLP-1 receptor) administered to patients with type 2 diabetes has shown great promise, resulting in a loss of ~2 kg over 4 weeks and improved glycemic control (Poon et al., 2005). By contrast, ghrelin has the opposite effect of GLP-1: it increases food intake in a variety of species (Tschöp et al., 2000; Wren et al., 2001a; Wren et al., 2001b); hence, therapeutic efforts have focused on blocking ghrelin signaling by means of various receptor antagonists. In another parallel with mammalian systems, there is growing evidence of the regulation of specific food choices and overall feeding behavior by various *Drosophila* peptides (Nässel and Winther, 2010).

Neuropeptide F, which is the *Drosophila* ortholog of mammalian neuropeptide Y, was identified based on a radioimmunoassay using a peptide from the corn earworm (Brown et al., 1999). It was subsequently found to regulate feeding behavior and multiple stress responses (Wu et al., 2003; Xu et al., 2010). *Drosophila* also produces a shorter neuropeptide F, referred to as sNPF. Overexpression of sNPF increases both food consumption and overall body size, whereas loss of sNPF decreases food intake (Lee et al., 2004). Later studies revealed that the size increase associated with sNPF overexpression is due largely to its effect on insulin secretion. sNPF regulates the release of DILPs from IPCs. Subsequently, the increased circulating levels of DILPs systemically increase growth and metabolism (Lee et al., 2008b). Importantly, sNPF mutants phenocopy most of the phenotypes associated with dampened insulin signaling, including lifespan extension and elevation of circulating sugar levels, confirming that a major effect of sNPF signaling in *Drosophila* is to augment insulin signaling (Lee et al., 2008b).

Interestingly, although alteration of feeding behavior usually produces a net increase or decrease in total caloric intake, there are instances where there is no net change in total calories consumed. For instance, disruption of the leucokinin pathway at the level of the peptide or receptor in *Drosophila* results in the consumption of large meal portions. However, this is associated with a decreased frequency of meal consumption, resulting in no net change in total caloric intake relative to wild-type flies (Al-Anzi et al., 2010). In addition, activation of neurons expressing allatostatin A suppresses starvation-induced feeding without affecting triglyceride or glucose levels (Hergarden et al., 2012).

There are other relatively less-well-characterized peptides that might also modulate feeding behavior, physiology and metabolism. As a case in point, loss of the brain-secretory polypeptide prothoracicotropic hormone (PTTH) delays larval development, extends the feeding period and leads to a concomitant increase in body size (McBrayer et al., 2007). In addition, expression of the cardioactive peptide corazonin decreases during periods of stress, and ablation of corazonin neurons confers tolerance to multiple stresses and alters energy stores (Lee et al., 2008a; Zhao et al., 2010). Intriguingly, sex peptide, a peptide present in seminal fluid introduced into female flies by copulation, increases feeding behavior in females (Carvalho et al., 2006), and diuretic hormone 31 regulates the passage of food along the midgut (LaJeunesse et al., 2010). The neuropeptide hugin regulates feeding behavior and is delivered by axonal projections that reach the pharyngeal muscles, which are required for food uptake (Melcher and Pankratz, 2005). In addition, some DILP7-producing neurons innervate the adult hindgut and regulate feeding behavior in response to nutrient availability (Miguel-Aliaga et al., 2008; Cognigni et al., 2011). Furthermore, it has been shown that, in addition to their established role in DILP secretion, IPCs also secrete drososulfakinins, which act as a satiety signal by regulating both food choice and intake (Söderberg et al., 2012). These observations underscore the complexity of appetite regulation through peptidergic signaling and emphasize the need to study this phenomenon in a combinatorial context. Future studies will clarify the exact molecular mechanisms underpinning the action of these less-characterized peptides, and how their modulation affects lifespan and metabolic homeostasis.

## Insights from *Drosophila* models associated with aberrant energy homeostasis

### Obesity and high-fat-diet models

Most of the major metabolic enzymes in mammals are conserved in *Drosophila*. For instance, the genes that regulate lipid uptake, transport, storage and mobilization are all well conserved (Oldham and Hafen, 2003; Kim and Rulifson, 2004; Baker and Thummel, 2007; Leopold and Perrimon, 2007; Trinh and Boulianne, 2013). Given the similarities between fly and human metabolism, there have been attempts to unravel as-yet-unknown mechanisms that regulate high-fat-diet (HFD)-induced obesity in *Drosophila* (Birse et al., 2010). In one such study, several parallels between human and fly obesity were observed (Birse et al., 2010). For instance, similar to humans, flies accumulate lipids in a dose-dependent manner, become obese when raised on an HFD and can accumulate excess dietary fat in non-adipose tissue. An interesting observation in flies, which could have etiological implications for cardiovascular diseases in humans, is that flies fed an HFD develop cardiomyopathy. Importantly, systemic inhibition of the TOR pathway disrupts the accumulation of excess fats and prevents the high-fat-induced impairment of cardiac function. In addition, cardiac function was preserved in flies fed an HFD even when downregulation of TOR was restricted only to the heart. Similar observations were made with flies overexpressing FOXO or lipase specifically in the heart. It has therefore been proposed that targeted inhibition of the TOR pathway might be a viable therapeutic approach for ameliorating the effect of obesity on cardiac function (Table 2).

**Table 2.**
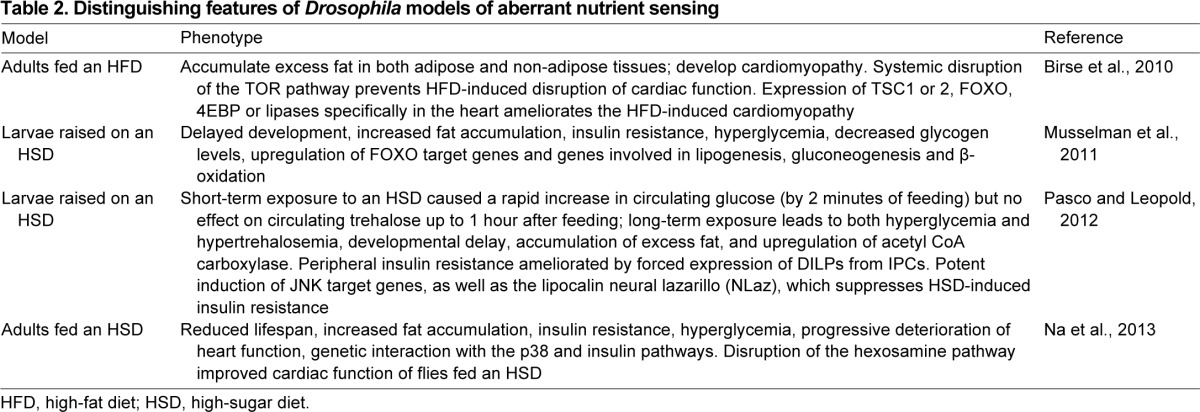
Distinguishing features of *Drosophila* models of aberrant nutrient sensing

Although the accumulation of excess calories from HFDs is a major environmental factor linked to obesity, there is growing evidence for a strong hereditary component as well. In this regard, genetic screens to uncover the polygenic basis of obesity will hold great promise. Attempts to find unknown genes and pathways that regulate obesity have focused on transgenic and knockout mouse models modeling human obesity, quantitative trait loci (QTL) from animal breeding experiments and linkage analyses (Rankinen et al., 2006). Although such approaches have their merits, a major pitfall is that they preclude large-scale analyses owing to their prohibitive costs. In a particularly illuminating example of how *Drosophila* genetics can be used to bridge this gap, a genome-wide transgenic RNAi screen was performed in *Drosophila* to uncover modulators of fat body formation (Pospisilik et al., 2010). One of the genes identified was *hedgehog* (*hh*), which was subsequently found to play a key role in the determination of brown versus white adipocyte cell fate in mice (Pospisilik et al., 2010). Importantly, more than 60% of the candidate genes from the screen were conserved from *Drosophila* to humans, and numerous genes previously known to play crucial roles in mammalian lipid metabolism, such as enzymes that regulate glucose or sterol metabolism and membrane lipid biosynthesis, scored positively in the screen. Strikingly, many of the candidate genes for regulating lipid metabolism had no previously ascribed biological functions; thus, further characterization of these candidate genes is likely to reveal additional regulatory strategies for fat metabolism in mammals.

Another promising approach for elucidating conserved novel pathways or genes associated with obesity is to combine studies in *Drosophila* with those in a mammalian system. In one such example, a screen was performed in *Drosophila* to identify genes that either increased or decreased triglyceride levels (Dohrmann, 2004). Out of ~10,000 mutants screened, 200 candidate genes were found to alter total triglyceride content. In parallel experiments to identify chromosomal loci that are susceptible to obesity, the authors analyzed offspring from New Zealand obese (NZO) mice outcrossed to lean mouse strains (Kluge et al., 2000; Plum et al., 2000; Plum et al., 2002). They intensely pursued a specific chromosomal region that contained several genes that were also present in the candidate list obtained from the *Drosophila* screen. Further studies resulted in the characterization of one of these ‘high-confidence’ genes – CG17646 in *Drosophila* – which is the mammalian ortholog of ABCG1 (an ATP-binding cassette transporter). More extensive analyses using knockout mouse models revealed that disruption of ABCG1 expression impeded the rate at which mice gained weight over a 12-week period. This was associated with a reduction in total mass of adipose tissue and a significantly reduced size of the adipocytes (Buchmann et al., 2007). Thus, the combined analyses of data from flies and mice resulted in the elucidation of a previously unrecognized role of ABCG1 in the regulation of metabolic homeostasis.

In summary, because the various models of obesity in *Drosophila* recapitulate the predominant features of obesity in humans, the stage is set for elaborate genetic screens that will help decipher additional causative factors of obesity. In particular, additional studies to further dissect the effect of cardiac lipotoxicity in *Drosophila* could open up novel therapeutic opportunities.

### Diabetes and high-sugar diet models

A number of research groups have established models to study diabetes and the effect of high-sugar diets (HSDs) in *Drosophila* (Musselman et al., 2011; Pasco and Léopold, 2012; Na et al., 2013). For instance, larvae fed a high-calorie diet develop hyperglycemia, a hallmark of diabetes in humans, which is typically scored in fruit flies as an increase in both hemolymph glucose and trehalose, the primary circulating sugar in this organism (Musselman et al., 2011). It is noteworthy that the hyperglycemia was more severe when flies were fed an HSD, with the extent of hyperglycemia similar to what is observed in insulin-resistant flies or flies with their IPCs ablated (Rulifson et al., 2002; Song et al., 2010). Additional experiments revealed that larvae fed an HSD also displayed peripheral insulin resistance (Musselman et al., 2011). Thus, all the major features of diabetes were recapitulated in this *Drosophila* larval model (Table 2). In a remarkable parallel with mammalian systems, insulin-resistant animals have higher amounts of stored fat as detected by an increase in total triglyceride levels and size of lipid droplets in their adipocytes (Musselman et al., 2011).

A second *Drosophila* larval diabetic model also revealed that an HSD causes peripheral insulin resistance (Table 2) and that forced secretion of DILPs can overcome the effect of the HSD (Pasco and Léopold, 2012). Unquestionably, the outstanding feature of this report was that the peripheral insulin resistance triggered by the HSD was mediated by the lipocalin NLaz (neural lazarillo). NLaz is an ortholog of the vertebrate lipocalins – lipocalin 2 and retinol binding protein 4 (RBP4) – which modulate peripheral insulin resistance and have been associated with metabolic homeostasis (Yang et al., 2005; Graham et al., 2006). However, the precise roles of these mammalian lipocalins had been controversial. For instance, although RBP4 levels correlated with insulin resistance in some type-II diabetes patients (Gavi et al., 2007; Kim et al., 2012), other reports have shown the opposite (von Eynatten et al., 2007; Al-Daghri et al., 2009). Thus, the authors sought to clarify the role of NLaz by using their model of HSD-induced insulin resistance. They found that, among the three fly orthologs of lipocalin, only one (i.e. NLaz) was robustly induced in HSD-fed larvae. Subsequently, they observed that a mutant *NLaz* allele, or fat-body-restricted knockdown of *NLaz*, could rescue the metabolic defects of the HSD-fed larvae. Thus, in addition to showing a correlation between HSD-induced insulin resistance and NLaz expression, the therapeutic potential of disrupting lipocalin function in type-II diabetes patients was established.

Adult flies fed an HSD develop severe structural and functional alterations of the heart (Table 2), as well as essentially all the phenotypes observed in the larval model (Musselman et al., 2011; Na et al., 2013). Defects in fly heart function were first manifest as arrhythmias, which then progressed to fibrillations and asystolic periods (Na et al., 2013). Previous reports in mice had shown that the hexosamine biosynthetic pathway might influence the extent of pathogenesis of type-II diabetes (Liu et al., 2000; Kaneto et al., 2001; Brownlee, 2005). Accelerating the rate of flux of hexosamine in cardiomyocytes promotes hyperglycemia-induced apoptosis (Frustaci et al., 2000). Accordingly, activation of the hexosamine synthetic pathway in *Drosophila* hearts resulted in aberrant heart function (Na et al., 2013). Interestingly, disrupting the activities of two enzymes that regulate hexosamine biosynthesis suppressed the sugar-induced cardiac dysfunction, raising the possibility that disrupting hexosamine biosynthesis might be a plausible therapeutic option for curtailing diet-induced cardiomyopathy (Na et al., 2013).

Altogether, the HSD diabetic models in *Drosophila* faithfully capture the salient features of type-II diabetes. Given that perturbations of specific biosynthetic or signaling pathways that had previously been associated with type-II diabetes (such as hexosamine biosynthesis and NLaz) notably impact disease progression, the future is ripe for the identification of druggable targets that can impede the progression of this debilitating condition using well-designed genetic screens in *Drosophila*.

## Intersection between nutrient sensing, metabolic homeostasis and aging

Nutrient-sensing pathways have been linked to aging in multiple organisms. It is known that dietary restriction extends lifespan in multiple organisms and at least delays the age-dependent decline in function of primates such as monkeys (Fontana et al., 2010; McKiernan et al., 2011; Anderson and Weindruch, 2012). In humans, it delays the age of onset of diseases such as cancer, diabetes and heart disease (Anderson and Weindruch, 2012). Additionally, reduced insulin or TOR signaling extends lifespan in yeast, the roundworm *Caenorhabditis elegans*, *Drosophila* and mice (Fontana et al., 2010; Kenyon, 2010; Evans et al., 2011; Johnson et al., 2013).

**Fig. 1.**
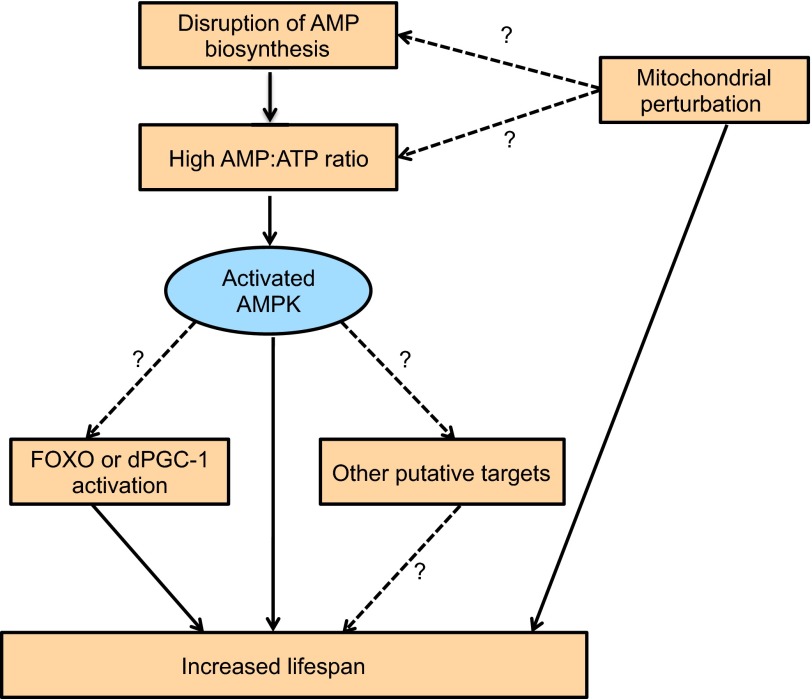
**AMPK-mediated lifespan extension and putative interactors.** Solid arrows denote established connections, whereas broken arrows with question marks highlight possible interactors. Rising AMP levels lead to activation of AMPK, which ultimately promotes an extension to lifespan; the targets of AMPK are not fully known but might include FOXO and dPGC-1 (see text for details).

Another nutrient-responsive kinase activated under conditions of energy deprivation is the AMP-activated protein kinase (AMPK) (Hardie et al., 2003). When AT P levels fall, AMP levels rise, resulting in the activation of AMPK, which in turn orchestrates a complex signaling network that restores cellular energy homeostasis (Hardie et al., 2012) (Fig. 1). Evidence of the beneficial effects of AMPK activation in mammalian systems is based on the fact that metformin (an AMPK activator) is a potent anti-diabetic agent (Zhou et al., 2001). In addition, studies in *C. elegans* have highlighted the fact that the ratio of AMP:ATP levels is predictive of lifespan and that AMPK activation promotes longevity (Apfeld et al., 2004; Curtis et al., 2006; Greer et al., 2007; Mair et al., 2011). It has recently been shown that AMPK promotes the secretion of AKH when flies are subjected to metabolic stress. Disruption of AMPK function specifically in AKH-secreting cells recapitulates many of the phenotypes associated with AKH ablation and results in increased lifespan (Braco et al., 2012). Moreover, targeted expression of AMPK in either adult flight muscles or adipose tissue extends lifespan (Stenesen et al., 2013).

Surprisingly, mutations in several genes controlling AMP biosynthesis, which intuitively should reduce AMP levels and consequently the ratio of AMP:ATP, also increased lifespan. The authors resolved this apparent paradox by demonstrating that mutations in genes for AMP biosynthesis actually increased AMP:ATP ratios and activated AMPK (Stenesen et al., 2013). However, in an additional study where flies were fed metformin, an increase in lifespan was not observed (Slack et al., 2012). Further studies are required to resolve whether the disparity is due to nonspecific effects of metformin or a requirement for AMPK activation in specific tissues instead of globally. Additionally, although it remains to be seen how AMPK activation mechanistically engages a pro-longevity cue, studies in other organisms might provide some valuable hints. For instance, the lifespan-promoting effect of AMPK in *C. elegans* is partially dependent on the well-established anti-aging transcription factor FOXO (Greer et al., 2007). Furthermore, the growing list of factors activated by AMPK in mammalian systems includes several pro-longevity proteins such as mTOR and PGC-1α (Hardie et al., 2012). In this regard, it is interesting to note that overexpression of the *Drosophila* ortholog of PGC-1 (dPGC-1 or Spargel) in stem and progenitor cells of the adult fly gut extends lifespan. In addition, forced expression of dPGC-1 in either larvae or adults is sufficient to increase mitochondrial activity (Rera et al., 2011). It is unclear whether dPGC-1 mediates some of the longevity-enhancing effects of AMPK (Fig. 1); nevertheless, further studies in *Drosophila*, focusing on how disruption of various putative downstream targets of AMPK affect AMPK-mediated lifespan extension, should help resolve the AMPK targets responsible for lifespan extension.

Given that AMPK activation increases lifespan, it is worth speculating whether reduced ATP levels and subsequent AMPK activation could account for the pro-longevity effect of mild mitochondrial perturbation – a phenomenon that has been consistently observed in both *C. elegans* and *Drosophila* (Lee et al., 2003; Copeland et al., 2009). However, long-lived flies with perturbed mitochondrial function do not consistently display reduced ATP levels (Copeland et al., 2009). This raises the possibility that other metabolites that become elevated in response to mitochondrial perturbation should be investigated for their possible lifespan-promoting effects. Interestingly, some *Drosophila* mitochondrial mutants increase reactive oxygen species (ROS) production, which in turn activates signaling cascades to elicit specific developmental or cell cycle responses (Owusu-Ansah et al., 2008; Owusu-Ansah and Banerjee, 2009). Nevertheless, whether elevated levels of ROS are required for lifespan extension in flies is still an open question.

## Concluding remarks

Here, we have highlighted some of the advances made in modeling nutrient sensing and metabolic homeostasis in *Drosophila*. Interestingly, a recent biochemical resource uncovered more than 400 different lipids that vary in expression during the life cycle of *Drosophila* (Guan et al., 2013), and a narrowly tuned fructose receptor was shown to function as a nutrient sensor in the brain (Miyamoto et al., 2012). In addition, it was recently shown that a *Drosophila* adiponectin receptor in IPCs regulates circulating trehalose levels (Kwak et al., 2013); however, the precise ligand (the *Drosophila* functional ortholog of adiponectin) that signals through the receptor remains to be identified. Thus, it appears that much remains to be uncovered in this highly burgeoning field. An important limitation of metabolic studies in *Drosophila* is that the main circulating sugar is trehalose, instead of glucose as in humans. Interestingly, trehalose has potent antioxidant activity (Alvarez-Peral et al., 2002) – hence, its elevation in response to various signaling pathways might impact an organism’s response to stress, thereby producing phenotypes some of which might not extrapolate to those of humans. Similar concerns arise over the fact that flies are unable to synthesize cholesterol (Gilbert et al., 2002). In addition, future studies will have to more thoroughly dissect the neural circuits that regulate feeding behavior, especially determining how such circuits are related to others that regulate alternative behaviors. Some recent reports have broached this subject: specific interneurons acting downstream of metabolic cues have been shown to control the decision to feed (Flood et al., 2013), and a pair of interneurons in the ventral nerve cord regulates the choice between locomotion and feeding (Mann et al., 2013). An additional drawback at the moment is that most *Drosophila* models of metabolism have only served to recapitulate phenotypes that are well-established in mammalian systems. Although this admittedly has served to validate the use of *Drosophila* to study mammalian metabolism, future studies will have to ‘set the pace’ by uncovering novel signaling modules or therapeutic strategies for countering diseases associated with aberrant metabolic homeostasis in humans.
